# Effects of photobiomodulation therapy on functional recovery, angiogenesis and redox status in denervated muscle of rats

**DOI:** 10.31744/einstein_journal/2021AO6001

**Published:** 2021-09-13

**Authors:** Jéssica Junia Aparecida Cardoso Nascimento, Alex Sander Dias Machado, Giovanna Moura Lamas Della-Santa, Danielle Cristina Fernandes, Marcílio Coelho Ferreira, Gustavo Augusto Pereira Machado, Bruna Carolina Garcia Chaves, Karine Beatriz Costa, Etel Rocha-Vieira, Murilo Xavier Oliveira, Thais Peixoto Gaiad, Ana Paula Santos

**Affiliations:** 1 Universidade Federal dos Vales do Jequitinhonha e Mucuri DiamantinaMG Brazil Universidade Federal dos Vales do Jequitinhonha e Mucuri, Diamantina, MG, Brazil.

**Keywords:** Muscles, Crush injuries, Phototherapy, Oxidative stress, Vascular endothelial growth factors, Muscle strength, Rats, Wistar

## Abstract

**Objective:**

To evaluate the effects of photobiomodulation therapy in redox status, angiogenesis marker – vascular endothelial growth factor – and in the functional recovery in denervated muscle.

**Methods:**

A total of 32 female Wistar rats underwent a crush injury and were randomly divided into four groups: Light Emitting Diode Group 2 and Control Group 2 (muscle collected 2 days after injury), and Light Emitting Diode Group 21 and Control Group 21 (muscle collected 21 days afterinjury). Light Emitting Diode Group 2 and Light Emitting Diode Group 21 received two and ten light emitting diode applications (630±20nm, 9J/cm^2^, 300mW), respectively, and the Control Group 2 and Control Group 21 did not receive any treatment. The function was evaluated by grasping test at four moments (pre-injury, 2, 10 and 21 post-injury days). The flexor digitorum muscle was collected for analysis of immunolocalization of vascular endothelial growth factor and redox parameters.

**Results:**

Functional improvement was observed at the second and tenth post-injury day in treated groups compared to control (p<0.005). The muscle tissue of treated groups presented higher immunohistochemical expression of vascular endothelial growth factor. Photobiomodulation therapy decreased the oxidative damage to lipid in Light Emitting Diode Group 2 compared to Control Group 2 (p=0.023) in the denervated muscle.

**Conclusion:**

Photobiomodulation therapy accelerated the functional recovery, increased angiogenesis and reduced lipid peroxidation in the denervated muscle at 2 days after injury.

## INTRODUCTION

The human peripheral nervous system responds to axonal injuries through the active regeneration; however, the treatment of injured peripheral nerves is far from being optimal.^(^^1^^)^ Patients suffer from severe impairment of their quality of life due to decline in the functionality, and their treatment is still associated with high socioeconomic costs.^(^^1^^,^^2^^)^ For functional recovery it is necessary to prevent the denervation-related skeletal muscle atrophy.^(^^3^^)^

Photobiomodulation therapy (PBMT), red (600nm to 700nm) or infrared (770nm to 1200nm) light spectrum,^(^^4^^)^ is widely investigated and used for nerve regeneration purposes.^(^^1^^)^ Despite the considerable number of studies about PBMT action in nerve regeneration, most of them focus on performing morphometric, electrophysiological and functional analyses,^(^^5^^,^^6^^)^ which leads to scarcity of studies in the literature about PBMT mechanisms of action on nerve regeneration and denervated muscle.

Redox imbalance is one of the main causes of neural or target-organ damage after injuries. In addition, it can lead to delayed functional recovery of the peripheral nerve.^(^^7^^)^ Nerve tissues are particularly sensitive to free radicals and appear to inhibit the antioxidant system when they are injured.^(^^7^^)^ Denervated muscle showed a significant increase in the mitochondrial generation of hydrogen peroxide, associated to progressive loss of muscle mass of the tibialis anterior muscle, from 7 up to 21 days following denervation.^(^^8^^)^ Mitochondria in the denervated tibialis anterior muscle of mice showed increased peroxide generation by 3 days after transection.^(^^9^^)^ These authors attested the presence of recent denervation in aging muscle fibers increased reactive oxygen species (ROS) generation by mitochondria of innervated fibers nearby and denervated fibers.

The vascular endothelial growth factor (VEGF) is a multifunctional cytokine, known to promote axonal regeneration and protect muscle fibers from degeneration.^(^^3^^,^^10^^)^ It is also known that the increased vascular permeability associated with neovascularization, promoted by VEGF, are crucial events for the tissue under repair, because the increased blood supply to the injured cells allows oxygen and a variety of cytokines and growth factors to reach the injury site.^(^^10^^)^

Photobiomodulation therapy affects the redox state of injured muscle tissue, and increases the gene expression of antioxidant enzymes in stressed cells,^(^^11^^)^ enables nitric oxide (NO) photodissociation from cytochrome c oxidase in mitochondria, and improves cell respiration and adenosine triphosphate production.^(^^12^^)^ In addition, PBMT decreases the production of ROS^(^^4^^,^^11^^)^ that, despite playing an essential role in the redox signaling pathway necessary to enable important biological events, can damage molecules that are essential to several cell processes.^(^^13^^)^

Photobiomodulation therapy increases immunnohistochemical expression of VEGF expression in muscle tissue.^(^^14^^)^ Several studies point that the VEGF interacts with skeletal muscle satellite cells and promotes regeneration after trauma.^(^^3^^,^^15^^)^ Furthermore, VEGF has been shown to have a myogenic and antiapoptotic effect,^(^^3^^)^ and appears to be able to improve the restoration of muscle strength, and reduce the amount of connective tissue after traumatic injury.^(^^15^^)^

Thus, PBMT becomes a good option to help controlling damages to injured nerve and denervated muscle tissues. Obviously, PBMT has other action mechanisms besides the modification of the cell redox state and expression of growth factors.^(^^4^^,^^12^^)^ However, these mechanisms should certainly be taken into consideration.

## OBJECTIVE

To investigate the effect of photobiomodulation therapy using light emitting diode on the redox state and the immunohistochemical expression of vascular endothelial growth factor in denervated muscles at two different moments after the injury, as well as to analyze the influence of this therapy on functional recovery.

## METHODS

### Animals and surgical procedure

The present study was approved by the Institutional Ethics Committee on Animal Research, under protocol 46/2015 and was carried out in Laboratory of Animal Experimentation of the Physical Therapy Department of *Universidade Federal dos Vales do Jequitinhonha e Mucuri* between September 2017 and December 2018.

A total of 32 female Wistar rats (age: 7 weeks; weight: 140 to 190g) were used. They were kept in boxes (four animals in each) placed in an air-conditioned room (22°C to 23°C), where they had access to water/feed *ad libitum* , and were subjected to 12-hour light cycles.

**Figure 1:**
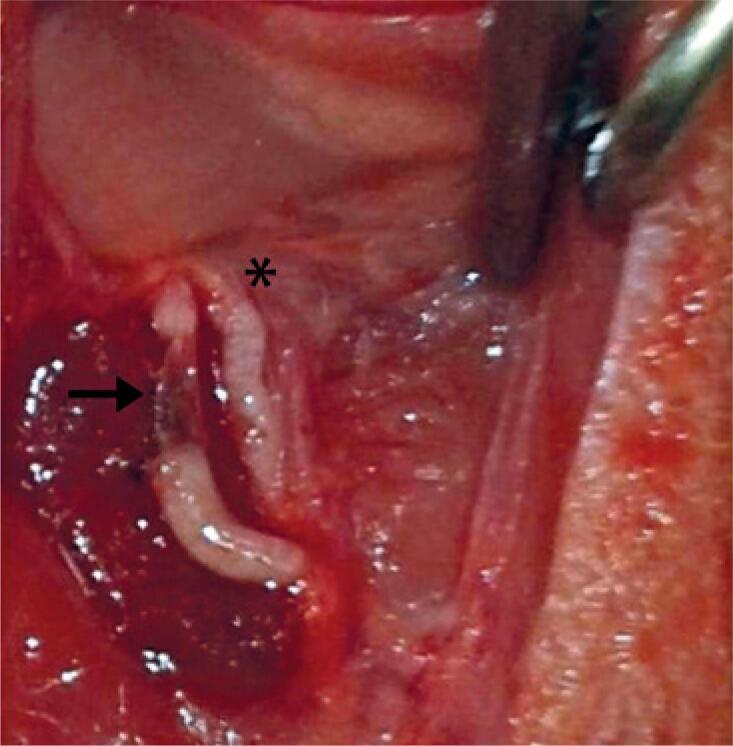
Median nerve crush, 10mm above the elbow. Arrow indicates the injury site

The rodents were intraperitoneally anesthetized with xylazine 2% and ketamine 10% (0.1mL/100g). They were placed in dorsal decubitus position to enable performing trichotomy and asepsis of the site; the right median nerve was exposed 10mm above the elbow, and a crush injury was induced using standard hemostatic forceps, in which the second notch was utilized for maintaining the nerve crush. The same was maintained closed, during 2 minutes, to crush the nerve^(^^16^^)^ ( Figure 1 ). Subsequently, the skin was sutured with 4-0 thread and treated with chlorhexidine digluconate (Merthiolate, Gold Lab). After surgery, the animals were randomly divided into four groups: Light Emitting Diode Group 2 (LG2; eight animals were subjected to two transcutaneous applications of PBMT and were euthanized 48 hours after the injury induction); Control Group 2 (CG2; eight animals received no treatment and were euthanized 48 hours after the injury induction); Light Emitting Diode Group 21 (LG21; eight animals were subjected to ten PBMT applications and were euthanized 21 days after the injury induction); Control Group 21 (CG21; eight animals received no treatment and were euthanized 21 days after the injury induction).

### Photobiomodulation therapy

The parameters proposed for PBMT using light emitting diode (LED) device (Bios Therapy II, Bios^®^) were wavelength (630±20nm), optical output (300mW), energy density (9J/cm^2^), power density (0.3W/cm^2^), energy (9J), spot size (1cm^2^), treatment time (30 seconds), LED mode (continuous output), number of irradiation points (1), and total energy delivered (216J). The animals were manually immobilized, and the treatment was transcutaneously applied to the surgical site, at a single point. The beam angle was kept perpendicular (90°) to the irradiation surface.

The application of the first therapeutic procedure was right after surgery. The LG2 Group was subjected to the second and final application 24 hours later. The LG21 Group was subjected to ten applications (five applications, one per day, at 2-day intervals and, subsequently, other five applications).

### Functional test

The grasping test^(^^17^^)^ was applied to the right thoracic limb of the animals by two trained researchers, who were blind to the experimental group of each animal. One researcher gently lifted the rats by their tail and allowed them to grab a grid attached to an ordinary electronic scale. Next, he gently pulled it up, while the other researcher recorded the generated negative value. The contralateral thoracic limb of the animals was restrained with duct tape to prevent it from gripping the grid. The test was performed in four different experimental stages: pre-injury, 2, 10 and 21 post-injury days.

### Immunohistochemistry analysis

Primary polyclonal antibodies against VEGF (Novus Bio NB100527) 1:400 were applied on muscle sections. Sections were immersed in citric acid solution at 0.01M, pH 6.0 and submitted to 95°C for 30 minutes to antigenic recovery. Next, the blockade of endogenous peroxidase with hydrogen peroxide at 3% for 40 minutes was performed. Primary antibodies were applied and incubated for 20 hours in a damp chamber at 4°C. After three more rinses in buffered saline solution,^(^^18^^)^ secondary (Biotinylated/Dako) and tertiary (Streptavidin-HRF/Dako) antibodies were applied and incubated for 30 minutes, at room temperature (24°C). Immunohistochemistry analysis (IHC) reaction was developed with diaminobenzidine (DAB) (Sigma Co., S. Louis, USA) for 1 minute. In the negative control, the primary antibody was omitted, and all slides were counterstained with hematoxylin. All tissue photomicrographs for immunohistochemistry analyses were made under an optical microscope (Labomed^®^ LxPol), equipped with an Axio CAM HRc camera and Software Capture Pro 2.9.0.1.

### Analyses of redox state parameters

Samples were collected based on the protocol applied to each group of animals: two and 21 days after injury induction. Animals were pre-anesthetized with 0.1mL/100g of a mixture comprising ketamine 10% and xylazine 2%, after the application of the same aseptic and surgical conditions adopted in the initial procedure. The flexor digitorum muscle was fully removed. After this procedure, the removed muscle tissue were extensively washed in buffered saline solution (pH 7.2), weighed and stored in the freezer at −80°C, until biochemical analysis.

A Potter-Elvehjem (Corning) tissue grinder (kept in ice) was used to macerate the samples in ice-cold buffered saline solution (0.015M) at pH 7.4. The homogenate was divided into two aliquots, according to the herein performed assays. One aliquot was centrifuged at 5,000g, 4°C, for 5 minutes, and the supernatant was used to analyze thiobarbituric acid reactive substances (TBARS) and the non-enzymatic antioxidant capacity of the tissues. The sediment was used to measure the carbonyl derivative content in proteins. The other aliquot was centrifuged at 10,000g, 4°C, for 10 minutes, and the supernatant was used to evaluate the enzymatic antioxidant capacity of the tissues, by measuring the activity of enzymes, such as superoxide dismutase (SOD) and catalase (CAT).

The TBARS concentration,^(^^19^^)^ measured based on thiobarbituric acid reaction to malondialdehyde (MDA), was used to determine lipid peroxidation. Sample aliquots of 0.15mL were added to 0.1mL sodium dodecyl sulfate (SDS; 8.1%), 0.25mL acetic acid (2.5M; pH 3.4) and 0.25mL thiobarbituric acid (0.8%). The mixture was homogenized and incubated at 95°C for 90 minutes. After this procedure was over muscle samples were cooled and centrifuged at 5,000g for 5 minutes, and 0.25mL of the supernatant was removed and placed on a 96-well flat-bottom plate for spectrophotometric reading at 532nm. Thiobarbituric acid reactive substances content was expressed in nmol MDA/mg of protein, based on the standard curve of known MDA concentrations. Measurements were carried out in duplicate.

The non-enzymatic antioxidant capacity of the samples was determined based on the Ferric Reducing Antioxidant Power (FRAP) method.^(^^20^^)^ To prepare the FRAP reagent, 25mL of sodium acetate buffer (0.3M, pH 3.6) were added to 2.5mL of tripyridyl triazine (TPTZ) (10mM TPTZ) and, then, to 2.5mL of iron chloride (FeCl_3_H_2_O −20 mM). Next, a 528μL aliquot of FRAP reagent was added with 72μL of homogenate. The mixture was homogenized and incubated in the dark at 37°C, for 30 minutes. Subsequently, samples were centrifuged at 300g, for 5 minutes, and the supernatant was spectrophotometrically analyzed (in duplicate), in microplate reader at 593nm. The total antioxidant capacity of the samples was determined based on the standard curve of known ferrous sulfate (FeSO4) concentrations and normalized based on the amount of protein in the sample; results were expressed in μM FeSO_4_/mg of protein.

The homogenate sediment of muscle tissue was suspended in 1mL of 50mM potassium phosphate buffer (pH 6.7) containing 1mM ethylenediaminetetraacetic acid (EDTA), and samples were divided into blank and test groups to enable measuring the carbonyl derivative concentrations in proteins.^(^^21^^)^ Trichloroacetic acid at 10% was added to all samples, which were centrifuged at 5,000g, at 4°C for 10 minutes; the supernatant was discarded. Next, 2,4-dinitrophenylhydrazine (10mM DNPH) diluted in 2mM hydrochloric acid was added to the test sediment. The blank was added with 2mM hydrochloric acid only. Samples were kept in the dark, at room temperature, for 30 minutes and vortex-homogenized every 15 minutes. Trichloroacetic acid at 10% was added to the samples, which were homogenized and centrifuged at 5,000g, 4°C, for 10 minutes. The supernatant was discarded and the sedimented material was washed in 1mL ethanol and ethyl acetate (1:1), and centrifuged two times, at 5,000g, 4°C, for 10 minutes. Finally, the sedimented material was dissolved in 6% SDS and centrifuged at 10,000g, 4°C, for 10 minutes. The supernatant was spectrophotometrically analyzed at 370nm, in a 96-well plate microplate reader, at 22,000M^-1^cm^-1^ DNPH molar absorption coefficient. The carbonyl derivative content in proteins was expressed in mmol of carbonyl derivatives per mg of protein (mmol/mg of protein); measurements were conducted in triplicate.

Superoxide dismutase activity was determined based on the inhibition of pyrogallol autoxidation.^(^^22^^)^ Samples of each tissue were added to potassium phosphate buffer (50mM, pH 8.2, 37°C). containing 1mM diethylenetriaminepentaacetic acid, and the reaction was triggered by the addition of 0.2mM pyrogallol (1,2,3-benzenetriol); the reading was spectrophotometrically performed in microplate reader at 420nm, 37°C, for 4 minutes. SOD activity (expressed in U/mg) was determined based on the enzyme's ability to inhibit pyrogallol autoxidation by 50%; measurements were performed in duplicate.

Catalase activity was determined based on hydrogen peroxide absorbance decrease at 240nm.^(^^23^^)^ A hydrogen peroxide solution at 0.3M was added to the samples, and the reaction deriving from its decomposition was spectrophotometrically monitored in quartz cuvettes at 240nm, 25°C, for 1 minute; measurements were performed in triplicate. Catalase activity was expressed in millimolar of H_2_O_2_ decomposed per minute per milligram of protein (ΔE/min/mg of protein).

### Statistical analysis

Statistical analysis was performed using GraphPad Prism 5 software. Morphometric and histological data are presented as mean±standard deviation (SD). Qualitative assessments of immunohistochemistry of muscle samples were analyzed by observing three sections from each one of the animals (n=8)/per group=4. The Shapiro-Wilk normality test was used. The *t* -test for independent samples was used to compare the variables of the redox state of denervated muscles and of the median nerve function. Differences were considered significant at p<0.05.

## RESULTS

Results of the functional analysis are described in table 1 . It is possible to observe that LG accelerated the functional return in the first two moments of evaluation (2 and 10 days post-injury). There was no significant difference in functional recovery between LG21 and CG21 on the 21^st^ post-injury day.

**Table 1:** Values obtained in the grasping functional test

Groups	Pre-Injury	2PL	10PL	21PL
LG2	234.40±46.40	1.88±2.59 *	-	-
CG2	211.40±42.50	0.00±0.00	-	-
LG21	183.80±37.01	6.25±2.31 *	39.38±20.43 *	140.00±16.04
CG21	178.10±52.09	1.87±2.58	14.38±5.63	163.80±45.02

* Indicates a significant difference between LG2 and CG2; and LG21 and CG21. Differences were considered significant at p<0.05 ( *t* -test for independent samples).

PL: post-injury: LG2: Light Emitting Diode Group 2; CG2: Control Group 2; LG21: Light Emitting Diode Group 21; CG21: Control Group 21.

**Figure 2:**
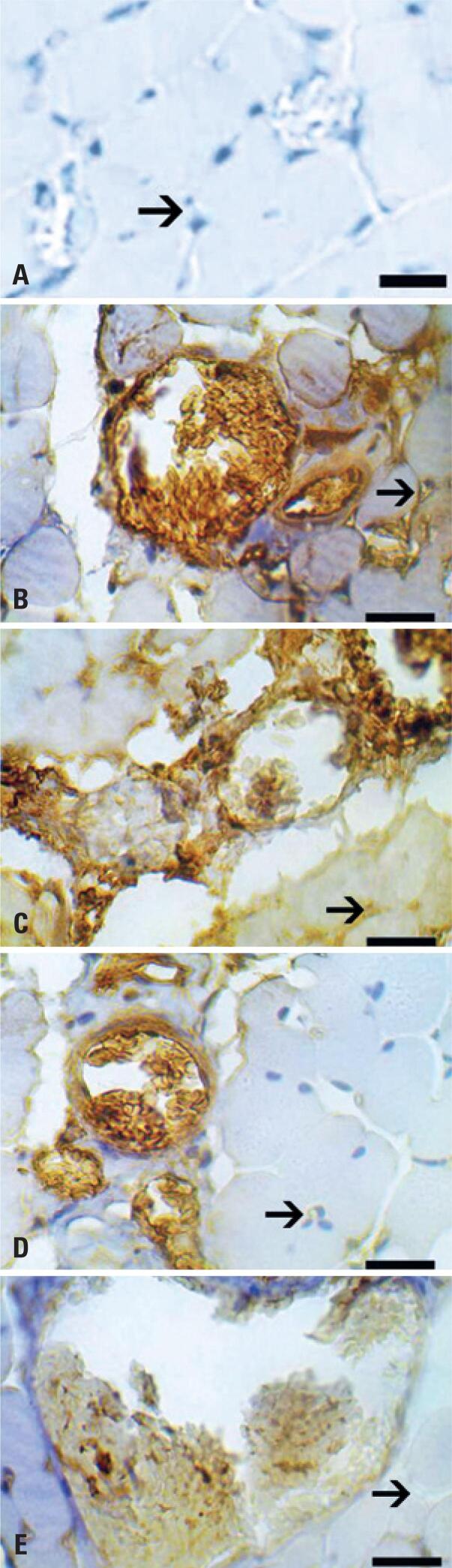
Photomicrography of immunohistochemistry of muscle sections. Vascular endothelial growth factor antibody. Dilution of 1:400. A) Negative control of reaction; B) Light Emitting Diode Group 2; C) Control Group 2; D) Light Emitting Diode Group 21; E) Control Group 21. Magnification=1000x, bar=10 μm. Arrows indicate angiogenesis areas on muscle

The immunohistochemical analysis of VEGF showed denervated muscles of treated groups expressed more VEGF staining than control ones. This expression was higher at 2 days group than 21 days ones. Denervated muscles of PBMT groups expressed also closer angiogenesis points/places when compared to controls ( Figure 2 ).

**Figure 3:**
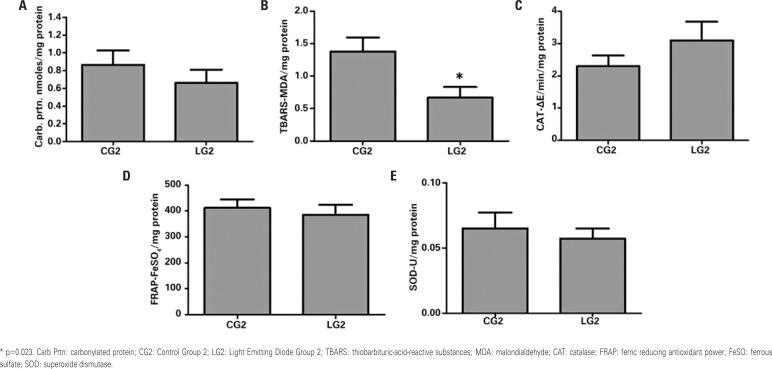
Redox state parameters in the denervated muscle on the second post-injury day in the control and light emitting diode groups. A) Carbonylated protein; B) Thiobarbituric-acid-reactive substances; C) Catalase; D) Ferric reducing antioxidant power; E) Superoxide dismutase

**Figure 4:**
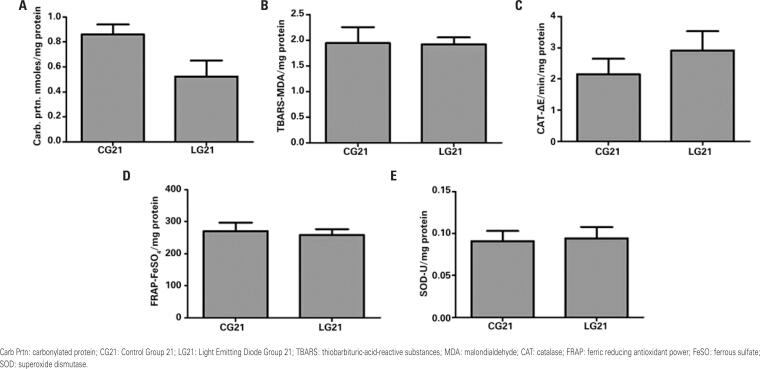
Redox state parameters in the denervated muscle on the 21^st^ day post-injury in the control and light emitting diode groups. A) Carbonylated protein; B) Thiobarbituric-acid-reactive substances; C) Catalase; D) Ferric reducing antioxidant power; E) Superoxide dismutase

Figure 3 shows the results of redox state biomarkers of denervated muscles in CG2 and LG2. Light Emitting Diode Group 2 presented lower TBARS concentration in denervated muscles than CG2 (p=0.023). Figure 4 shows data about biomarkers in denervated muscles of the groups euthanized 21 days after injury induction.

## DISCUSSION

The current study was the first to describe the action of PMBT on redox state biomarkers and VEGF immunnohistochemical expression in denervated muscles associated with functional responses. The results showed that the PBMT using the LED device after axonotmesis can increase VEGF immunnohistochemical expression and change redox state parameters, such as lipid peroxidation and oxidative damage to proteins, in denervated muscles.

Photobiomodulation therapy accelerated the functional recovery of the analyzed animals, since there were significant differences between the irradiated and control groups on the second and tenth post-injury days.

The grasping test adopted in the current study is objective and effective in demonstrating the day when the functional recovery began, as well as its progression.^(^^17^^,^^24^^)^ Studies conducted with rodents have already demonstrated that animals subjected to median nerve crushing, which remained untreated, began to flex their fingers on the eighth post-injury day, presented reduced strength on the 9^th^ day, as well as satisfactory improvement after the tenth day,^(^^17^^)^ and recovered full function 20-21 days after injury induction.^(^^25^^)^

The accelerated functional recovery after axonotmesis has already been described for PBMT using LED. The evaluation of the sciatic nerve function of Wistar rats (the nerve was harmed with hemostatic forceps for 30 seconds) was based on the sciatic functional index; rats treated with LED presented better functional outcomes at the seventh, 14^th^ and 21^st^ post-injury days.^(^^6^^)^ The starting date and the number of LED irradiations were similar to this study. However, the parameters wavelength (940nm), power density (9.5mW/cm²) and energy density (4/cm²) were different.^(^^6^^)^ In addition, the nerve compression time was different, and this interferes in the results.^(^^24^^)^

The median nerve was selected because it presents lesser incidence of contractures and autotomy, which are often seen in sciatic nerve injuries; these processes can affect the evaluation of nerve regenerative parameters and end up not preserving animals’ well-being. The distance between the median nerve and the target organs is small, and it allows faster reinnervation; besides, most nerve injuries in humans happen in the upper limbs.^(^^24^^)^

Among the neurotrophic factors involved in nerve regeneration, the VEGF has essential function.^(^^26^^)^ The VEGF is known to promote axonal regeneration, neurogenesis, neuroprotection, stimulation of glial growth, functional reinnervation and neovascularization.^(^^3^^,^^10^^)^ In the denervated muscle, local applications of VEGF attenuate atrophy.^(^^3^^)^ Like the denervation-induced capillary regression may be associated with downregulation of VEGF, the PBMT could be a choice for increasing the VEGF expression.^(^^14^^)^

The increase in VEGF expression found in the denervated muscle in this study was not observed in the sciatic nerve crushed with laser (780nm, 30mW and 15J/cm²).^(^^10^^)^ The PBMT parameters, among them the light spectrum, and the crushed nerves interfere in the results and were different between studies.^(^^5^^)^ Regarding the light spectrum, endothelial cells in culture that underwent laser irradiation in the infrared and red spectrum responded significantly to the application of the red spectrum. There was no significant difference in the number of endothelial cells irradiated by infrared laser.^(^^27^^)^

The higher imunohistochemical expression of VEGF observed in the LED groups, associated with the lower lipid and protein damage found, demonstrated the beneficial effect that neovascularization provides for tissue recovery. We can also highlight the qualitative difference in immunohistochemical expression of VEGF, between 2 and 21 days, in LED groups. Such difference demonstrates that just as the vessels are neoformed, induced by the increased oxygen demand of the injured cells; when the inflammation recedes, decreases the number of vessels, with the degeneration of endothelial cells and vascular regression.^(^^28^^)^

It is known that PBMT has positive effects on denervated muscles, since it reduces muscle atrophy and improves muscle functions.^(^^2^^)^ However, no reports about the action of PBMT on oxidative parameters and growth factors in denervated muscles were found in the literature. The current study has found lesser lipid damage in the denervated muscle tissues of LED-irradiated groups. This outcome corroborated with the decreased lipid peroxidation after PBMT application in stressed muscles.^(^^11^^)^

Crush injuries cause redox imbalance in nerve tissues. Increased ROS and lipid peroxidation, as well as decreased antioxidant activity, were already described in this injury model.^(^^7^^)^ According to Pigna et al.,^(^^29^^)^ although denervated muscles presented increased ROS, there was no increase in carbonyl groups and in lipid peroxidation. In addition, oxidative stress was not responsible for muscle damage and atrophy. On the other hand, Sayir et al.,^(^^30^^)^ observed that both neurotmesis and axonotmesis presented increased lipid peroxidation and reduced antioxidant CAT, SOD, glutathione peroxidase and carbonic anhydrase activity in denervated muscles. Abruzo et al.,^(^^31^^)^ also found ROS production and lipid peroxidation in denervated muscles, as well as abundant cytoprotective and antioxidant RNA proteins, such as CAT and SOD, and increased glutathione S-transferase and glutathione peroxidase activity.

The PBMT application site (on the injury, in the current study) should be taken into consideration at the time of analysis of light effects, since different results have already been described after nerve crushing, depending on the light application site.^(^^32^^)^ Besides, other factors such as irradiation frequency, energy and power density, emission and irradiation time, should be taken into consideration in PBMT protocols.^(^^5^^)^

Since innervation is an important trophic factor that regulates the functional and structural integrity of muscle tissues, muscle preservation, or decrease of the progressive muscle degeneration are the major challenges in nerve injury cases.^(^^31^^)^ The current study showed that PBMT can be used for muscle and functional preservation purposes after nerve injury. However, it is necessary to conduct further studies focused on evaluating the action of PBMT on the redox state, and growth factors in injured nerves and denervated muscles.

## CONCLUSION

The photobiomodulation therapy using LED (630nm; 9J/cm^2^; 300mW) accelerated the functional recovery, increased immunnohistochemical expression of vascular endothelial growth factor, and reduced lipid peroxidation in denervated muscles at 2 days after injury induction.
