# Supplementary material for: Effects of photobiomodulation therapy on functional recovery, angiogenesis and redox status in denervated muscle of rats
Source: Einstein (Sao Paulo). 2021 Sep 13;19:eAO6001. doi: 10.31744/einstein_journal/2021AO6001 (PMC8439560; doi:10.31744/einstein_journal/2021AO6001)
Supplement: Translation

# Translation: Efeitos da terapia de fotobiomodulação na recuperação funcional, na angiogênese e no estado redox em músculo desnervado de ratos

**Authors:** Jéssica Junia Aparecida Cardoso Nascimento, Alex Sander Dias Machado, Giovanna Moura Lamas Della-Santa, Danielle Cristina Fernandes, Marcílio Coelho Ferreira, Gustavo Augusto Pereira Machado, Bruna Carolina Garcia Chaves, Karine Beatriz Costa, Etel Rocha-Vieira, Murilo Xavier Oliveira, Thais Peixoto Gaiad, Ana Paula Santos

**Affiliations:** 1 DiamantinaMG Brasil Universidade Federal dos Vales do Jequitinhonha e Mucuri, Diamantina, MG, Brasil.

**Keywords:** Músculos, Lesões por esmagamento, Fototerapia, Estresse oxidativo, Fatores de crescimento do endotélio vascular, Força muscular, Ratos Wistar

## Abstract

**Objetivo:**

Avaliar os efeitos da terapia de fotobiomodulação no estado redox, no marcador de angiogênese – fator de crescimento endotelial vascular – e na recuperação funcional do músculo desnervado.

**Métodos:**

Um total de 32 ratas Wistar foi submetido a uma lesão por esmagamento e dividido aleatoriamente em quatro grupos: Grupo Diodo Emissor de Luz 2 e Grupo Controle 2 (músculo coletado 2 dias após a lesão), além do Grupo Diodo Emissor de Luz 21 e do Grupo Controle 21 (músculo coletado 21 dias após a lesão). Grupo Diodo Emissor de Luz 2 e Grupo Diodo Emissor de Luz 21 receberam duas e dez aplicações de diodo emissor de luz (630±20nm, 9J/cm^2^e 300mW), respectivamente, e Grupo Controle 2 e Grupo Controle 21 não receberam tratamento. A função foi avaliada pelo teste de preensão em quatro momentos (pré-lesão, 2, 10 e 21 dias após a lesão). O músculo flexor dos dedos foi coletado para análise dos parâmetros redox e da imunolocalização do fator de crescimento endotelial vascular.

**Resultados:**

Houve melhora funcional no segundo e décimo dia pós-lesão nos grupos tratados em comparação aos controles (p<0,005). O tecido muscular dos grupos tratados apresentou maior expressão imuno-histoquímica do fator de crescimento endotelial vascular. A terapia de fotobiomodulação diminuiu o dano oxidativo aos lipídeos no Grupo Diodo Emissor de Luz 2 comparado ao Grupo Controle 2 (p=0,023) no músculo desnervado.

**Conclusão:**

A terapia de fotobiomodulação acelerou a recuperação funcional, aumentou a angiogênese e reduziu a peroxidação lipídica no músculo desnervado 2 dias após a lesão.

## INTRODUÇÃO

O sistema nervoso periférico humano responde a lesões axonais por meio de regeneração ativa; porém, o tratamento de nervos periféricos lesados está longe de ser ideal.^(^^1^^)^Os pacientes têm grave comprometimento de sua qualidade de vida devido à redução na funcionalidade, e o tratamento ainda está associado a altos custos socioeconômicos.^(^^1^^,^^2^^)^Para a recuperação funcional é necessário prevenir a atrofia do músculo esquelético relacionada à desnervação.^(^^3^^)^

A terapia de fotobiomodulação (TFBM), espectro de luz vermelha (600nm a 700nm) ou infravermelho (770nm a 1.200nm),^(^^4^^)^é amplamente investigada e utilizada para fins de regeneração nervosa.^(^^1^^)^Apesar do número considerável de estudos sobre a ação da TFBM na regeneração nervosa, a maioria deles se concentra na realização de análises morfométricas, eletrofisiológicas e funcionais,^(^^5^^,^^6^^)^o que leva à escassez de estudos na literatura sobre os mecanismos de ação da TFBM na regeneração nervosa e no músculo desnervado.

O desequilíbrio redox é uma das principais causas de danos neurais ou em órgãos-alvo após lesões. Além disso, pode resultar em uma recuperação funcional retardada do nervo periférico.^(^^7^^)^Os tecidos nervosos são particularmente sensíveis aos radicais livres e parecem inibir o sistema antioxidante quando são lesados.^(^^7^^)^O músculo desnervado apresentou aumento significativo na geração mitocondrial de peróxido de hidrogênio, associado à perda progressiva de massa muscular do músculo tibial anterior, de 7 a 21 dias após a desnervação.^(^^8^^)^As mitocôndrias do músculo tibial anterior desnervado de camundongos mostraram aumento da geração de peróxido 3 dias após a transecção.^(^^9^^)^Esses autores verificaram que a presença de desnervação recente nas fibras musculares em envelhecimento aumentou a geração de espécies reativas de oxigênio (ROS - *reactive oxygen species* ) pela mitocôndria de fibras inervadas próximas e fibras desnervadas.

O fator de crescimento endotelial vascular (VEGF) é uma citocina multifuncional, que sabidamente promove a regeneração axonal e protege as fibras musculares de degeneração.^(^^3^^,^^10^^)^Sabe-se também que o aumento da permeabilidade vascular associada à neovascularização, promovida pelo VEGF, é um evento crucial para o tecido em reparo, porque o aumento do suprimento de sangue para as células lesadas permite que oxigênio e várias citocinas e fatores de crescimento cheguem ao local da lesão.^(^^10^^)^

A TFBM afeta o estado redox do tecido muscular lesado, aumenta a expressão gênica de enzimas antioxidantes em células estressadas,^(^^11^^)^permite a fotodissociação do óxido nítrico (NO) da citocromo c oxidase na mitocôndria e melhora a respiração celular e a produção de trifosfato de adenosina.^(^^12^^)^Além disso, a TFBM diminui a produção de ROS,^(^^4^^,^^11^^)^que, apesar de desempenhar papel essencial na via de sinalização redox necessária para possibilitar eventos biológicos importantes, pode danificar moléculas essenciais a diversos processos celulares.^(^^13^^)^

A TFBM aumenta a expressão imuno-histoquímica da expressão do VEGF no tecido muscular.^(^^14^^)^Vários estudos apontam que o VEGF interage com as células satélite do músculo esquelético e promove a regeneração após o trauma.^(^^3^^,^^15^^)^Além disso, foi demonstrado que o VEGF tem ação miogênica e antiapoptótica,^(^^3^^)^parecendo ser capaz de melhorar a restauração da força muscular e reduzir a quantidade de tecido conjuntivo após lesão traumática.^(^^15^^)^

Assim, a TFBM torna-se uma boa opção para auxiliar no controle de danos ao nervo lesado e aos tecidos musculares desnervados. Obviamente, a TFBM possui outros mecanismos de ação, além da modificação do estado redox celular e da expressão de fatores de crescimento.^(^^4^^,^^12^^)^Entretanto, esses mecanismos certamente devem ser levados em consideração.

## OBJETIVO

Investigar o efeito da terapia de fotobiomodulação com diodo emissor de luz sobre o estado redox e a expressão imuno-histoquímica do fator de crescimento endotelial vascular em músculos desnervados em dois momentos distintos após a lesão, bem como analisar a influência dessa terapia na recuperação funcional.

## MÉTODOS

### Animais e procedimento cirúrgico

O presente estudo foi aprovado pelo Comitê de Ética em Pesquisa Animal da instituição, sob protocolo 46/2015, e foi realizado no Laboratório de Experimentação Animal do Departamento de Fisioterapia da Universidade Federal dos Vales do Jequitinhonha e Mucuri entre setembro de 2017 e dezembro de 2018.

Foram utilizadas 32 ratas Wistar fêmeas (7 semanas de idade; 140 a 190g de peso). Os animais foram acondicionados em caixas (quatro animais em cada) e colocados em sala com ar condicionado (22°C a 23°C), onde tiveram acesso a água/ração *ad libitum* , e foram submetidos a ciclos de luz de 12 horas.

Os roedores foram anestesiados intraperitonealmente com xilazina 2% e cetamina 10% (0,1mL/100g). Foram colocados em decúbito dorsal para possibilitar a realização da tricotomia e assepsia do local; o nervo mediano direito foi exposto 10mm acima do cotovelo, e uma lesão por esmagamento foi induzida usando uma pinça hemostática padrão, em que o segundo entalhe foi utilizado para manter o esmagamento do nervo. O mesmo foi mantido fechado, durante 2 minutos, para esmagamento do nervo^(^^16^^)^( Figura 1 ). A seguir, a pele foi suturada com linha 4-0 e tratada com digluconato de clorexidina (Merthiolate, Gold Lab). Após a cirurgia, os animais foram divididos aleatoriamente em quatro grupos: Grupo Diodo Emissor de Luz 2 (GL2; oito animais foram submetidos a duas aplicações transcutâneas de TFBM e sacrificados 48 horas após a indução da lesão); Grupo Controle 2 (GC2; oito animais não receberam tratamento e foram sacrificados 48 horas após a indução da lesão); Grupo Diodo Emissor de Luz 21 (GL21; oito animais foram submetidos a dez aplicações de TFBM e sacrificados 21 dias após a indução da lesão) e Grupo Controle 21 (CG21; oito animais não receberam tratamento e foram sacrificados 21 dias após a indução da lesão).

**Figura 1:** Esmagamento do nervo mediano 10mm acima do cotovelo. A seta indica o local da lesão

### Terapia de fotobiomodulação

Os parâmetros propostos para TFBM utilizando dispositivo de diodo emissor de luz (LED; Bios Therapy II, Bios^®^) foram comprimento de onda (630±20nm), potência óptica (300mW), densidade de energia (9J/cm^2^), densidade de potência (0,3W/cm^2^), energia (9J), tamanho do ponto (1cm^2^), tempo de tratamento (30 segundos), modo LED (saída contínua), número de pontos de irradiação (1) e energia total emitida (216J). Os animais foram imobilizados manualmente, e o tratamento foi aplicado por via transcutânea no sítio cirúrgico, em um único ponto. O ângulo do feixe foi mantido perpendicular (90°) à superfície de irradiação.

A aplicação do primeiro procedimento terapêutico foi logo após a cirurgia. O GL2 foi submetido à segunda e última aplicação 24 horas depois. O GL21 foi submetido a dez aplicações (cinco aplicações, sendo uma por dia, com intervalos de 2 dias e, posteriormente, outras cinco aplicações).

### Teste funcional

O teste de preensão^(^^17^^)^foi aplicado no membro torácico direito dos animais por dois pesquisadores treinados, que desconheciam o grupo experimental de cada animal. Um pesquisador gentilmente erguia os ratos pela cauda e permitia que pegassem uma grade presa a uma balança eletrônica comum. Em seguida, puxava-o com cuidado, enquanto o outro pesquisador registrava o valor negativo gerado. O membro torácico contralateral dos animais era preso com fita adesiva para evitar que agarrasse a grade. O teste foi realizado em quatro diferentes etapas experimentais: pré-lesão, 2, 10 e 21 dias pós-lesão.

### Análise imuno-histoquímica

Anticorpos policlonais primários contra VEGF (Novus Bio NB100527) 1:400 foram aplicados em cortes de músculo. Os cortes foram imersos em solução de ácido cítrico a 0,01M, com pH de 6,0, e submetidos à recuperação antigênica de 95°C, por 30 minutos. Em seguida, foi realizado o bloqueio da peroxidase endógena com peróxido de hidrogênio a 3% por 40 minutos. Os anticorpos primários foram aplicados e incubados por 20 horas em uma câmara úmida a 4°C. Após mais três lavagens em solução salina tamponada,^(^^18^^)^anticorpos secundários (Biotinilado/Dako) e terciários (Streptavidina-HRF/Dako) foram aplicados e incubados por 30 minutos, em temperatura ambiente (24°C). A reação de análise imuno-histoquímica foi revelada com diaminobenzidina (DAB; Sigma Co., S. Louis, Estados Unidos) por 1 minuto. No controle negativo, o anticorpo primário foi omitido, e todas as lâminas foram contrastadas com hematoxilina. Todas as fotomicrografias de tecidos para análise de imuno-histoquímica foram feitas em microscópio óptico (Labomed^®^LxPol), equipado com câmera Axio CAM HRc e Software Capture Pro 2.9.0.1.

### Análise dos parâmetros de estado redox

As amostras foram coletadas com base no protocolo aplicado a cada grupo de animais: 2 e 21 dias após a indução da lesão. Os animais foram pré-anestesiados com 0,1mL/100g de uma mistura de cetamina 10% e xilazina 2%, após a aplicação das mesmas condições assépticas e cirúrgicas adotadas no procedimento inicial. O músculo flexor dos dedos foi totalmente removido. Após esse procedimento, os tecidos musculares retirados foram extensivamente lavados em solução salina tamponada (pH de 7,2), pesados e armazenados em freezer a −80°C, até a análise bioquímica.

Um triturador de tecido Potter-Elvehjem (Corning), mantido em gelo, foi usado para macerar as amostras em solução salina tamponada gelado (0,015M) a pH de 7,4. O homogenato foi dividido em duas alíquotas, de acordo com os ensaios realizados. Uma alíquota foi centrifugada a 5.000g, 4°C, por 5 minutos, e o sobrenadante foi utilizado para analisar as substâncias reativas ao ácido tiobarbitúrico (TBARS) e a capacidade antioxidante não enzimática dos tecidos. O sedimento foi usado para medir o teor de derivados de carbonila nas proteínas. A outra alíquota foi centrifugada a 10.000g, 4°C, por 10 minutos, e o sobrenadante foi utilizado para avaliar a capacidade antioxidante enzimática dos tecidos, medindo a atividade de enzimas, como superóxido dismutase (SOD) e catalase (CAT).

A concentração de TBARS,^(^^19^^)^medida com base na reação do ácido tiobarbitúrico ao malondialdeído (MDA), foi usada para determinar a peroxidação lipídica. Amostras de alíquotas de 0,15mL foram adicionadas a 0,1mL de dodecilsulfato de sódio (SDS; 8,1%), 0,25mL de ácido acético (2,5M; pH de 3,4) e 0,25mL de ácido tiobarbitúrico (0,8%). A mistura foi homogeneizada e incubada a 95°C durante 90 minutos. Após o término desse procedimento, as amostras de músculo foram resfriadas e centrifugadas a 5.000g por 5 minutos, e 0,25mL do sobrenadante foi removido e colocado em uma placa de fundo plano de 96 poços para leitura espectrofotométrica a 532nm. O conteúdo de TBARS foi expresso em nmol MDA/mg de proteína, com base na curva padrão de concentrações conhecidas de MDA. As medições foram realizadas em duplicata.

A capacidade antioxidante não enzimática das amostras foi determinada com base no método do poder antioxidante redutor férrico (FRAP).^(^^20^^)^Para preparar o reagente FRAP, 25mL de tampão de acetato de sódio (0,3M, pH de 3,6) foram adicionados a 2,5mL de tripiridil triazina (TPTZ; TPTZ 10 mM) e, a seguir, a 2,5mL de cloreto de ferro (FeCl_3_H_2_O −20mM). Em seguida, uma alíquota de 528μL de reagente FRAP foi adicionada com 72μL de homogenato. A mistura foi homogeneizada e incubada no escuro a 37°C, durante 30 minutos. Posteriormente, as amostras foram centrifugadas a 300g, por 5 minutos, e o sobrenadante analisado por espectrofotometria (em duplicata) em leitor de microplacas a 593nm. A capacidade antioxidante total das amostras foi determinada com base na curva padrão de concentrações conhecidas de sulfato ferroso (FeSO4) e normalizada com base na quantidade de proteína na amostra; os resultados foram expressos em μM FeSO_4_/mg de proteína.

O sedimento homogenato do tecido muscular foi suspenso em 1mL de tampão de fosfato de potássio 50mM (pH de 6,7) contendo ácido etilenodiaminotetracético 1mM (EDTA), e as amostras foram divididas em grupos branco e de teste, para permitir a medição das concentrações de derivados de carbonila nas proteínas.^(^^21^^)^Foi adicionado ácido tricloroacético a 10% a todas as amostras, que foram centrifugadas a 5.000g, a 4°C, por 10 minutos; o sobrenadante foi descartado. Em seguida, foi adicionada 2,4-dinitrofenilhidrazina (DNPH; 10mM) diluída em ácido clorídrico (2mM) ao sedimento de teste. O branco foi adicionado com apenas 2mM de ácido clorídrico. As amostras foram mantidas no escuro, em temperatura ambiente, por 30 minutos, e homogeneizadas em vórtice, a cada 15 minutos. Foi adicionado ácido tricloroacético 10% às amostras, que foram homogeneizadas e centrifugadas a 5.000g, 4°C, por 10 minutos. O sobrenadante foi descartado, e o material sedimentado foi lavado em 1mL de etanol e acetato de etila (1:1) e centrifugado duas vezes, a 5.000g, 4°C, por 10 minutos. Finalmente, o material sedimentado foi dissolvido em SDS a 6% e centrifugado a 10.000g, 4°C, por 10 minutos. O sobrenadante foi analisado por espectrofotometria a 370nm, em leitor de microplacas de 96 poços, com coeficiente de absorção molar DNPH 22.000 M^-1^cm^-1^. O teor de derivados de carbonila nas proteínas foi expresso em mmol de derivados de carbonila por mg de proteína (mmol/mg de proteína); as medições foram realizadas em triplicado.

A atividade do SOD foi determinada com base na inibição da autoxidação de pirogalol.^(^^22^^)^Amostras de cada tecido foram adicionadas a tampão de fosfato de potássio (50mM; pH de 8,2; 37°C), contendo 1mM de ácido dietilenotriaminapentaacético, e a reação foi desencadeada pela adição de 0,2mM pirogalol (1,2,3-benzenotriol). A leitura foi realizada por espectrofotometria, em leitor de microplacas a 420nm, 37°C, por 4 minutos. A atividade de SOD, expressa em U/mg, foi determinada com base na capacidade da enzima de inibir a autoxidação de pirogalol em 50%; as medições foram realizadas em duplicata.

A atividade da CAT foi determinada com base na diminuição da absorbância do peróxido de hidrogênio a 240nm.^(^^23^^)^Uma solução de peróxido de hidrogênio a 0,3M foi adicionada às amostras, e a reação decorrente de sua decomposição foi monitorada por espectrofotometria, em cubetas de quartzo a 240nm, 25°C, por 1 minuto; as medições foram realizadas em triplicata. A atividade da CAT foi expressa em milimolar de H_2_O_2_decomposto por minuto por miligrama de proteína (ΔE/min/mg de proteína).

### Análise estatística

A análise estatística foi realizada com o *software* Prism 5 (GraphPad). Os dados morfométricos e histológicos são apresentados como média±desvio-padrão (DP). As avaliações qualitativas de imuno-histoquímica de amostras de músculo foram analisadas observando-se três cortes de cada um dos animais (n=8)/por grupo=4. Foi utilizado o teste de normalidade Shapiro-Wilk. O teste *t* para amostras independentes foi utilizado para comparar as variáveis do estado redox dos músculos desnervados e da função do nervo mediano. As diferenças foram consideradas significativas em p<0,05.

## RESULTADOS

Os resultados da análise funcional são descritos na tabela 1 . É possível observar que o GL acelerou o retorno funcional nos dois primeiros momentos de avaliação (2 e 10 dias pós-lesão). Não houve diferença significativa na recuperação funcional entre GL21 e GC21 no 21° dia pós-lesão.

**Tabela 1:** Valores obtidos no teste funcional de preensão

Grupos	Pré-lesão	2PL	10PL	21PL
GL2	234,40±46,40	1,88±2,59 *	-	-
GC2	211,40±42,50	0,00±0,00	-	-
GL21	183,80±37,01	6,25±2,31 *	39,38±20,43 *	140,00±16,04
GC21	178,10±52,09	1,87±2,58	14,38±5,63	163,80±45,02

* Indica diferença significativa entre o GL2 e o GC2; e o GL21 e o GC21. As diferenças foram consideradas significativas em p<0,05 (teste *t* para amostras independentes).

PL: pós-lesão; GL2: Grupo Diodo Emissor de Luz 2; GC2: Grupo Controle 2; GL21: Grupo Diodo Emissor de Luz 21; GC21: Grupo Controle 21.

A análise imuno-histoquímica do VEGF mostrou que os músculos desnervados dos grupos tratados expressaram mais coloração do VEGF do que os controles. Essa expressão foi maior no grupo de 2 dias do que no de 21 dias. Os músculos desnervados dos grupos TFBM também expressaram pontos/locais de angiogênese mais próximos quando comparados aos controles ( Figura 2 ).

**Figura 2:** Fotomicrografia imuno-histoquímica de cortes musculares. Anticorpo do fator de crescimento endotelial vascular. Diluição de 1:400. A) Controle negativo da reação; B) Grupo Diodo Emissor de Luz 2; C) Grupo Controle 2; D) Grupo Diodo Emissor de Luz 21; E) Grupo Controle 21. Aumento=1000x, barra=10μm. As setas indicam áreas de angiogênese no músculo

A figura 3 mostra os resultados dos biomarcadores do estado redox de músculos desnervados em GC2 e GL2. O GL2 apresentou menor concentração de TBARS em músculos desnervados do que o GC2 (p=0,023). A figura 4 mostra os dados dos biomarcadores nos músculos desnervados dos grupos sacrificados 21 dias após a indução da lesão.

**Figura 3:** Parâmetros de estado redox no músculo desnervado no segundo dia pós-lesão no grupo controle e no grupo com diodo emissor de luz. A) Proteína carbonilada; B) Substâncias reativas ao ácido tiobarbitúrico; C) Catalase; D) Poder antioxidante redutor férrico; E) Superóxido dismutase

**Figura 4:** Parâmetros do estado redox nos músculos desnervados no 21° dia pós-lesão nos grupos controle e de diodo emissor de luz. A) Proteína carbonilada; B) Substâncias reativas ao ácido tiobarbitúrico; C) Catalase; D) Poder antioxidante redutor férrico; E) Superóxido dismutase

## DISCUSSÃO

O presente estudo foi o primeiro a descrever a ação da TFBM nos biomarcadores do estado redox e na expressão imuno-histoquímica do VEGF em músculos desnervados associados a respostas funcionais. Os resultados mostraram que a TFBM usando o dispositivo LED após a axonotmese pode aumentar a expressão imuno-histoquímica do VEGF e alterar parâmetros do estado redox, como peroxidação lipídica e dano oxidativo às proteínas, em músculos desnervados.

A TFBM acelerou a recuperação funcional dos animais analisados, uma vez que houve diferenças significativas entre os grupos irradiados e os controles no segundo e no décimo dia pós-lesão.

O teste de preensão adotado no presente estudo é objetivo e eficaz em demonstrar o dia em que teve início a recuperação funcional, bem como sua progressão.^(^^17^^,^^24^^)^Estudos realizados com roedores já demonstraram que animais submetidos ao esmagamento do nervo mediano, que permaneceram não tratados, começaram a fletir os dedos no oitavo dia pós-lesão, apresentaram força reduzida no nono dia, bem como melhora satisfatória a partir do décimo dia,^(^^17^^)^e recuperaram a plena função 20 a 21 dias após a indução da lesão.^(^^25^^)^

A recuperação funcional acelerada após a axonotmese já foi descrita para TFBM usando LED. A avaliação da função do nervo ciático de ratos Wistar (o nervo foi lesado com pinça hemostática por 30 segundos) baseou-se no índice funcional do ciático; os ratos tratados com LED apresentaram melhores resultados funcionais no sétimo, 14^o^e 21^o^dias pós-lesão.^(^^6^^)^A data de início e o número de irradiações de LED foram semelhantes aos deste estudo. Porém, os parâmetros comprimento de onda (940nm), densidade de potência (9,5mW/cm²) e densidade de energia (4/cm²) foram diferentes.^(^^6^^)^Além disso, o tempo de compressão do nervo foi diferente, e isso interfere nos resultados.^(^^24^^)^

O nervo mediano foi selecionado por apresentar menor incidência de contraturas e autotomia, as quais costumam ocorrer nas lesões do nervo ciático. Esses processos podem afetar a avaliação dos parâmetros regenerativos do nervo e acabar não preservando o bem-estar dos animais. A distância entre o nervo mediano e os órgãos-alvo é pequena e permite uma reinervação mais rápida. Além disso, a maioria das lesões nervosas em humanos ocorre nos membros superiores.^(^^24^^)^

Dentre os fatores neurotróficos envolvidos na regeneração nervosa, o VEGF tem função essencial.^(^^26^^)^Sabe-se que ele promove regeneração axonal, neurogênese, neuroproteção, estimulação do crescimento glial, reinervação funcional e neovascularização.^(^^3^^,^^10^^)^No músculo desnervado, aplicações locais de VEGF atenuam a atrofia.^(^^3^^)^Como a regressão capilar induzida por desnervação pode estar associada à regulação negativa de VEGF, a TFBM pode ser uma escolha para aumentar a expressão do VEGF.^(^^14^^)^

O aumento da expressão do VEGF encontrado no músculo desnervado neste estudo não foi observado no nervo ciático esmagado com laser (780nm, 30mW e 15J/cm²).^(^^10^^)^Os parâmetros TFBM, entre eles o espectro de luz, e os nervos lesados interferem nos resultados e foram diferentes entre os estudos.^(^^5^^)^Em relação ao espectro de luz, as células endoteliais em cultura submetidas à irradiação a laser no infravermelho e no espectro vermelho responderam, de forma significativa, à aplicação do espectro vermelho. Não houve diferença significativa no número de células endoteliais irradiadas pelo laser infravermelho.^(^^27^^)^

A maior expressão imuno-histoquímica de VEGF observada nos grupos LED, associada ao menor dano lipídico e proteico encontrado, demonstrou o efeito benéfico que a neovascularização proporciona para a recuperação do tecido. Também é possível destacar a diferença qualitativa na expressão imuno-histoquímica do VEGF, entre 2 e 21 dias, nos grupos LED. Essa diferença demonstra que, assim como os vasos são formados recentemente, induzidos pelo aumento da demanda de oxigênio das células lesadas, quando a inflamação retrocede, diminui-se o número de vasos, com degeneração das células endoteliais e regressão vascular.^(^^28^^)^

A TFBM tem efeitos positivos nos músculos desnervados, pois reduz a atrofia muscular e melhora as funções musculares.^(^^2^^)^No entanto, não foram encontrados na literatura relatos sobre a ação da TFBM sobre os parâmetros oxidativos e fatores de crescimento em músculos desnervados. O estudo atual encontrou menor dano lipídico nos tecidos musculares desnervados de grupos irradiados com LED. Esse resultado corroborou a diminuição da peroxidação lipídica após a aplicação de TFBM em músculos estressados.^(^^11^^)^

Lesões por esmagamento causam desequilíbrio redox nos tecidos nervosos. Já foram descritos aumento de ROS e peroxidação lipídica, bem como diminuição da atividade antioxidante, nesse modelo de lesão.^(^^7^^)^Segundo Pigna et al.,^(^^29^^)^embora os músculos desnervados apresentassem aumento de ROS, não houve aumento nos grupos carbonila e em peroxidação lipídica. Além disso, o estresse oxidativo não foi responsável pelo dano muscular e pela atrofia. Por outro lado, Sayir et al.,^(^^30^^)^observaram que tanto a neurotmese quanto a axonotmese apresentaram aumento da peroxidação lipídica e redução da atividade antioxidante da CAT, do SOD, da glutationa peroxidase e da anidrase carbônica em músculos desnervados. Abruzo et al.,^(^^31^^)^também encontraram produção de ROS e peroxidação lipídica em músculos desnervados, além de abundantes proteínas de RNA citoprotetoras e antioxidantes, como CAT e SOD, e aumento da atividade da glutationa S-transferase e da glutationa peroxidase.

O local de aplicação da TFBM (na lesão, no presente estudo) deve ser levado em conta na hora de analisar os efeitos da luz, uma vez que diferentes resultados já foram descritos após o esmagamento do nervo, dependendo do local de aplicação da luz.^(^^32^^)^Além disso, outros fatores, como frequência de irradiação, densidade de energia e potência, emissão e tempo de irradiação, devem ser considerados nos protocolos de TFBM.^(^^5^^)^

Visto que a inervação é um importante fator trófico que regula a integridade funcional e estrutural dos tecidos musculares, a preservação muscular ou a diminuição da degeneração muscular progressiva são os principais desafios nos casos de lesão nervosa.^(^^31^^)^O presente estudo mostrou que a TFBM pode ser usada para fins de preservação muscular e funcional após lesão do nervo. No entanto, faz-se necessária a realização de mais estudos voltados para a avaliação da ação da TFBM sobre o estado redox e os fatores de crescimento em nervos lesados e músculos desnervados.

## CONCLUSÃO

A terapia de fotobiomodulação com LED (630nm; 9J/cm^2^; 300mW) acelerou a recuperação funcional, aumentou a expressão imuno-histoquímica do fator de crescimento endotelial vascular e reduziu a peroxidação lipídica em músculos desnervados 2 dias após a indução da lesão.
